# A Retrospective Cohort Outcome Study Comparing Gingival Margin and Buccal Sulcus Incisions for Mandibular Fracture Access

**DOI:** 10.3390/cmtr19030038

**Published:** 2026-08-31

**Authors:** Nivetha Saravanan, Millie Windram, Rory O’Connor

**Affiliations:** 1 Faculty of Dentistry, Oral & Craniofacial Sciences, King’s College London, Great Maze Pond, London SE1 9RT, UK; 2 School of Medicine, University of Nottingham, Derby Road, Nottingham NG7 2UH, UK; 3 Oral & Maxillofacial Surgery Department, Queen’s Medical Centre, Derby Road, Nottingham NG7 2UH, UK

**Keywords:** mandibular fracture, open reduction and internal fixation, intraoral incision, buccal sulcus incision, gingival margin incision, postoperative complication

## Abstract

(1) Background: The main intraoral incisions used in the operative management of mandibular fractures are gingival margin incisions and buccal sulcus incisions. The aim of this retrospective cohort study was to compare postoperative outcomes between incision types and guide future operative protocols. (2) Methods: Data was collected from Queen’s Medical Centre in Nottingham, a level I major trauma centre. BlueSpier was used to identify mandibular fracture patients operated on between January 2023 and January 2025. Digital health records were used to obtain patient demographics and postoperative outcomes. Primary outcome measures were postoperative infection and wound dehiscence. Secondary outcome measures were return to theatre and new numbness or paraesthesia. (3) Results: In this cohort, 138 patients underwent operative management for a mandibular fracture through an intraoral incision; 60 patients had a gingival margin incision and 78 patients had a buccal sulcus incision. No statistically significant difference was found between the gingival margin and buccal sulcus incision groups for the primary and secondary outcome measures. (4) Conclusions: Incision choice is dependent on mandibular fracture location and surgeon preference. Both incisions were used by most of the operating surgeons. This study was not designed as an equivalence or non-inferiority study. Further larger-scale studies are needed to identify superior outcomes of one approach.

## 1. Introduction

Fracture access is critical to the successful surgical treatment of mandibular fractures. Well-thought-through incisions permit good fracture access, enable fracture reduction, and when closed adequately, ensure soft tissue coverage of the fracture site and associated metalwork. Intraoral access to the fracture site is typically achieved using one of two incisions: the gingival margin (crevicular) incision or the buccal sulcus (vestibular) incision. A gingival margin incision is placed in the gingival crevice to raise a mucoperiosteal flap, often with relieving incisions at each end, to expose the upper and lower extent of the fracture line. In contrast, a buccal sulcus incision is placed in the mobile gingiva, 8–10 mm inferior to the mucogingival junction [1].

A randomised controlled trial (RCT) comparing incision types found that the gingival margin group had a statistically significant increase in postoperative mouth opening and a decrease in neurosensory disturbance compared with the buccal sulcus control group [2]. Nonetheless, the buccal sulcus incision remains the more commonly chosen approach and is technically simpler to perform, with easier plate placement [1]. Anecdotally, the buccal sulcus incision is also easier to close.

With a paucity of trials, which are of a small sample size, there is limited evidence to inform intraoral incision choice for mandibular fractures, which are commonly treated surgically. This retrospective cohort study aimed to identify postoperative outcomes for each incision type in the surgical management of acute mandible fractures in a level I Major Trauma Centre in the UK, with the intention of guiding fracture access in the future. It is acknowledged that a retrospective design has limitations in establishing superiority of one incision type. However, in light of the limited data in the existing published literature, a retrospective result is informative in offering surgeons institutional evidence at the point of care. By reporting on a combination of outcomes that include infection, return to theatre, wound dehiscence, and neurosensory deficit, this exploratory and descriptive study aims to align its outcome measures more closely to the complications that will influence decision making on incision choice in practice.

## 2. Materials and Methods

All patients with a mandibular fracture treated by open reduction and internal fixation (ORIF) that were accessed through an intraoral incision at the Queen’s Medical Centre in Nottingham, which is the East Midlands Major Trauma Centre, from January 2023 to January 2025 were included. This included mandibular fractures that had a comminution at the dentoalveolar level or inferior border of the mandible. Patients with isolated mandibular condylar fractures, fractures treated using an extraoral approach and those managed non-operatively were excluded.

**Figure 1:**
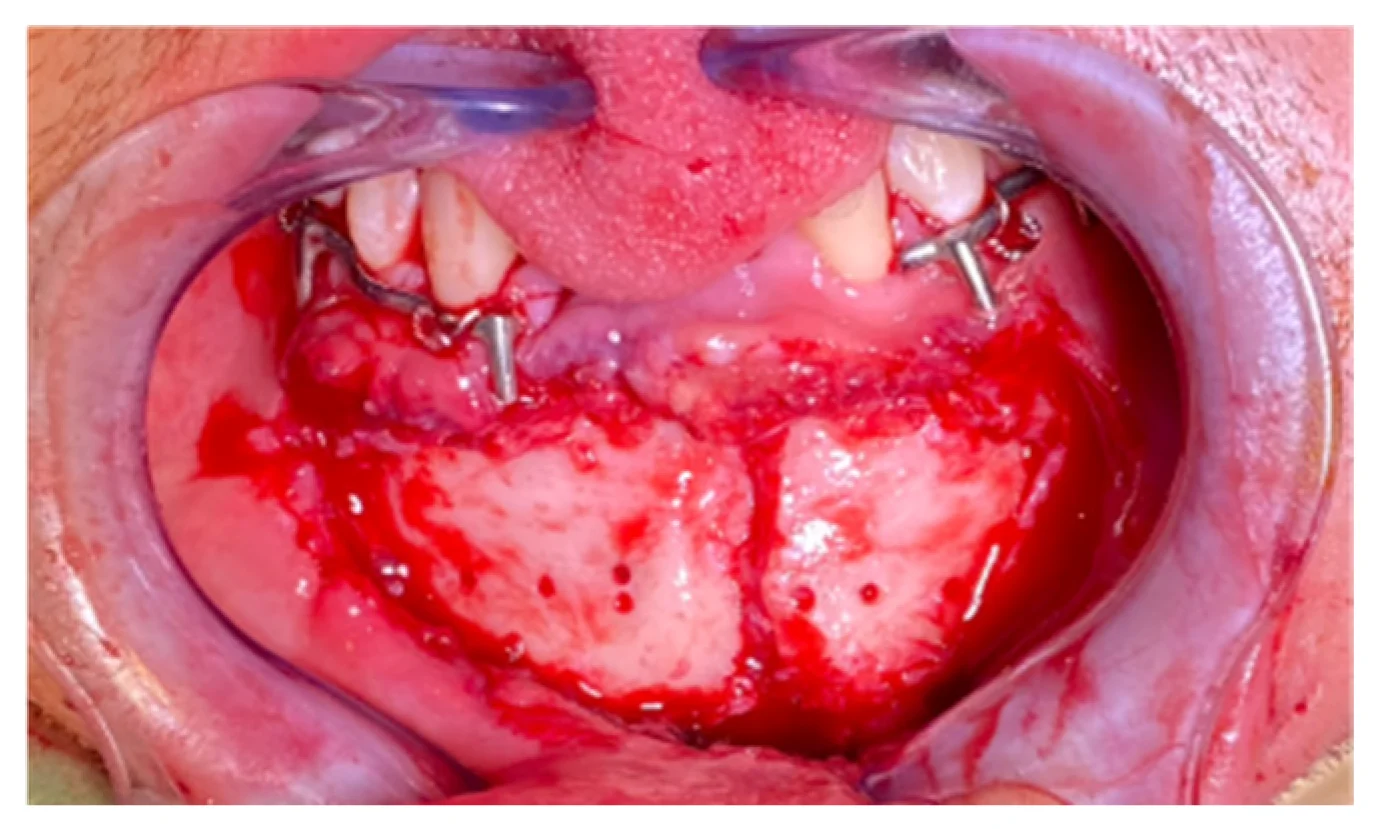
Intra-operative photograph showing a buccal sulcus incision for mandibular fracture access.

**Figure 2:**
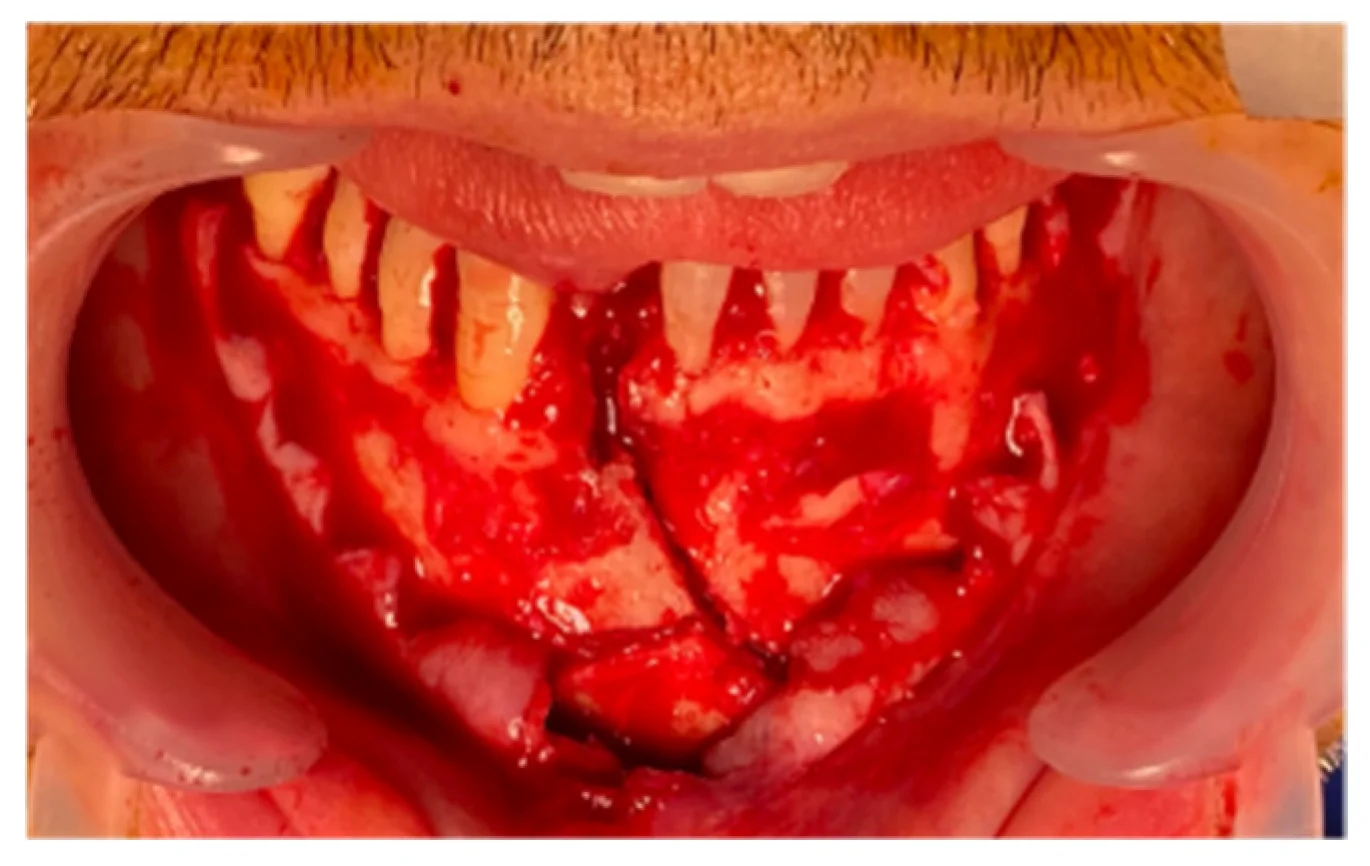
Intra-operative photograph showing a gingival margin incision for mandibular fracture access.

The hospital theatre management system, BlueSpier (Bluespier by Lanas©), was utilised to identify patients. The hospital digital health record (Unity Digital Health Record) was used to identify incision type, which was classified as either a buccal sulcus (Figure 1) or gingival margin (Figure 2) approach according to the operation notes, along with demographic data and postoperative outcomes. Incision type was assigned at the patient level from the operation note. All included patients received the same incision type when they had multiple included fractures. All outcome measures were recorded per patient rather than per fracture or per operative site.

Patients were followed up in the maxillofacial trauma clinic postoperatively and this documentation was used to identify the outcome measures. The observation period for all outcome measures was from the date of the operation to the date of the final documented trauma clinic review. Patients who did not attend any postoperative clinic reviews were planned to be retained and counted as having no recorded complications. Patients who attended some postoperative clinic reviews, but not all, had outcomes considered from those they did attend.

The primary outcome measures were postoperative infection and wound dehiscence. Postoperative infection was defined as a clinical diagnosis of a surgical site infection related to the fracture or fixation, recorded by the clinical team and requiring a therapeutic course of oral or intravenous antibiotics. Routine perioperative antibiotic prophylaxis was excluded. Wound dehiscence was defined as any documented separation of the intraoral mucosal wound margins, with or without exposure of underlying bone or metalwork.

The secondary outcome measures were return to theatre and new numbness, defined as anaesthesia, or paraesthesia indicative of mental nerve injury. Return to theatre was defined as any unplanned second operation related to the fracture fixation or infection. The new numbness or paraesthesia were documented postoperatively, through history and clinical examination, at the final postoperative clinic review. Neurosensory deficits recorded at presentation were regarded as preoperative numbness or paraesthesia, and were excluded from the new postoperative outcomes. Partial new numbness was defined as patient-reported reduced touch sensation and complete new numbness was defined as patient-reported absence of touch sensation. These were mutually exclusive subcategories. New paraesthesia was defined as a patient-reported “pins and needles” sensation, which could occur in combination with either numbness subcategory.

The null hypothesis was that there was no statistically significant difference in outcome measures between the gingival margin and buccal sulcus incision groups. Data on factors predisposing to infection, including smoking and diabetes, were also collected to examine the comparability of incision groups. Alcohol consumption and recreational drug use were recorded as binary variables from the social history section of the clerking documentation at presentation. They refer to self-reported current use by patients rather than a defined quantity or frequency. Where this information was not documented, the variable was recorded as absent. Additional variables were collected retrospectively for each patient on the level of comminution (none, at the dentoalveolar level, or at the inferior border of the mandible), the presence of a tooth within the fracture line, and whether this was extracted. The primary operator for each operation was also identified.

All statistical analyses were performed using IBM SPSS statistical software (version 31; IBM Corp., Armonk, NY, USA). Statistical significance was considered for values of *p* < 0.05. Demographic variables were compared between incision groups using binary logistic regression, with results reported as odds ratios (OR) with 95% confidence intervals (CI) and Wald test *p*-values. Variables with a zero-cell prevented the calculation of OR by standard logistic regression. In which case, Fisher’s exact test *p*-value was reported instead of the Wald test *p*-value. Categorical outcome measures were compared using the chi-squared test. Where cell frequencies fell below five in any cell of a 2 × 2 contingency table, Fisher’s exact test was substituted for the chi-squared test. Age was summarized as a median with interquartile range and compared with the Mann–Whitney U test, as it was not normally distributed.

## 3. Results

Of the 219 patient records obtained, 81 were removed due to having an extra-oral approach to surgical management. This left a total of 138 records for further analysis, of which 78 had a buccal sulcus incision, and 60 had a gingival margin incision. Fourteen surgeons were the primary operators in this cohort. Thirteen surgeons (seven consultants and six registrars) were the primary operators for the buccal sulcus incision group, and eleven (seven consultants and four registrars) for the gingival margin group. Ten surgeons used both approaches, and these accounted for 124/138 (90%) operations. A consultant was the primary operator in 61/78 (78%) of the buccal sulcus cases and in 50/60 (83%) of the gingival margin cases.

The median interval from injury to surgery was 2 days in the buccal sulcus group and 2 days in the gingival margin group. The minimum postoperative follow-up was 1.1 weeks, with a median of 4.7 weeks (range 1.1 to 35.3 weeks) in the buccal sulcus group. For the gingival margin group, the minimum postoperative follow-up was 1.3 weeks, with a median of 5.4 weeks (range 1.3 to 25.1 weeks). All included patients attended at least one postoperative review.

### 3.1. Demographics

**Figure 3:**
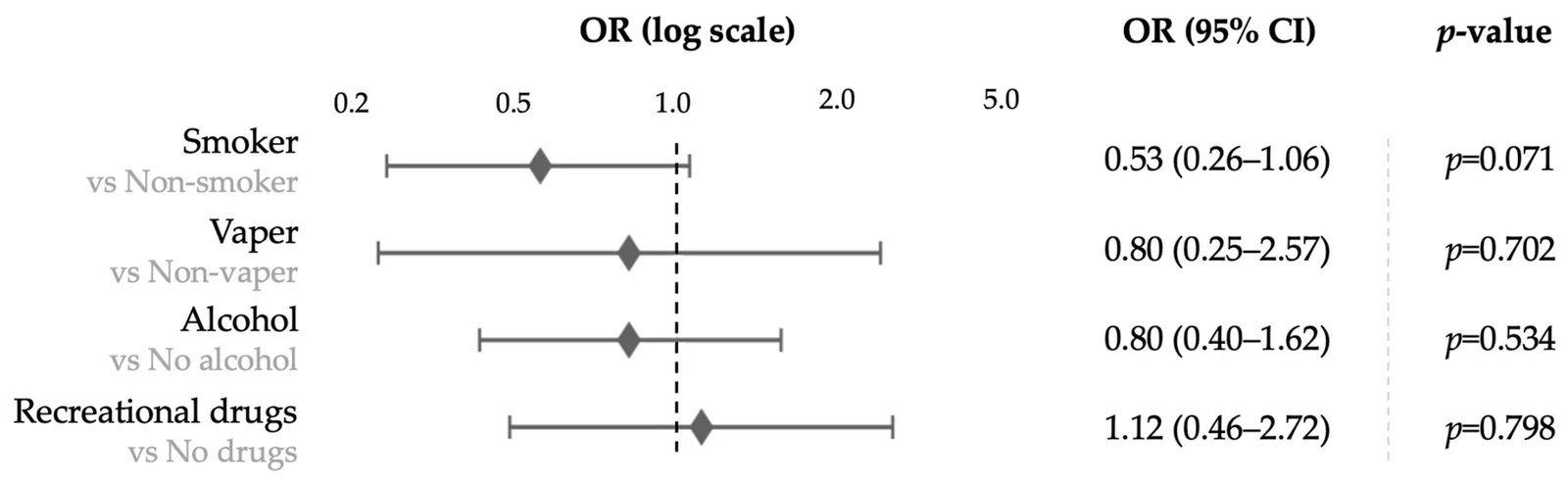
Forest plot of binary logistic regression analysis comparing demographic variables between buccal sulcus and gingival margin incision groups. Odds ratios (OR) with 95% confidence intervals (CI) are displayed on a log scale. OR > 1 is more prevalent in the gingival margin group; OR < 1 is more prevalent in the buccal sulcus group. Gender is excluded from the forest plot due to cell frequencies falling below five. Diabetes is excluded from the forest plot as a zero cell prevents OR estimation by standard logistic regression. Wald test *p*-values reported for all other variables.

The demographics of the patients included in this study and the results of logistic regression between incision groups are shown in Table 1 and as a forest plot in Figure 3. The cohort was predominantly male (93%, *n* = 129). No statistically significant differences were found between incision groups for gender (*p* = 0.502), vaping status (*p* = 0.702), alcohol consumption (*p* = 0.534), or recreational drug use (*p* = 0.798). Smoking status approached, but did not reach statistical significance (*p* = 0.071), with a higher proportion of smokers in the buccal sulcus group (49%) than the gingival margin group (33%). Cell frequencies fell below five for the gender comparison; therefore, Fisher’s exact test was applied. No patients in the buccal sulcus group had diabetes, compared to 5% of the gingival margin group. This prevented estimation of an odds ratio, and Fisher’s exact test was applied (*p* = 0.080).

### 3.2. Injury Location

The distribution of fracture locations differed significantly between the incision groups and is shown in Table 2. In patients presenting with a combination of mandibular fractures, each individual fracture was recorded as a separate entry. Therefore, the total number of fractures (*n* = 168) exceeds the total number of patients (*n* = 138). Angle fractures were more common in the buccal sulcus group (59 [60%] vs. 15 [22%]; *p* < 0.001), whereas parasymphysis fractures were more common in the gingival margin group (44 [64%] vs. 28 [28%]; *p* < 0.001). No significant differences were found for symphysis (*p* = 1.000), body (*p* = 0.930), or ramus (*p* = 0.569) fractures.

### 3.3. Fracture and Dental Characteristics

Fracture and dental characteristics in each incision group are shown in Table 3. Comminution at the dentoalveolar level was more common in the buccal sulcus group, whereas comminution at the inferior border of the mandible was more common in the gingival margin group. Most fractures were displaced in both groups, at 83% of the buccal sulcus group and 75% of the gingival margin group. A tooth was within the fracture line in the majority of cases in both incision groups, but was marginally more common in the buccal sulcus group (94% vs. 85%). Intra-operative dental extraction was more common in the buccal sulcus group (38% vs. 10%). A tooth was fractured by the injury in two patients in the buccal sulcus group and none in the gingival margin group.

### 3.4. Outcome Measures

The primary and secondary outcome measures are shown in Table 4. No statistically significant differences were identified between the buccal sulcus and gingival margin groups for the primary outcome measures of postoperative infection (7 [9%] vs. 5 [8%]; *p* = 0.893) with a chi-squared test and wound dehiscence (1 [1%] vs. 2 [3%]; *p* = 0.580) with Fisher’s exact test.

No statistically significant differences were found between groups for the secondary outcome measure of return to theatre for any reason (11 [14%] vs. 6 [10%]; *p* = 0.467) with the chi-squared test. On further analysis, no significant difference was found for return to theatre for infection (6 [8%] vs. 3 [5%]; *p* = 0.731) or for malocclusion (5 [6%] vs. 3 [5%]; *p* = 1.000) with Fisher’s exact test. Similarly, there was no statistically significant difference in new numbness or paraesthesia (25 [32%] vs. 23 [38%]; *p* = 0.442) with the chi-squared test. On further analysis, no significant difference was identified for partial new numbness (11 [14%] vs. 9 [15%]; *p* = 0.882), complete new numbness (6 [8%] vs. 5 [8%]; *p* = 0.890), or new paraesthesia (8 [10%] vs. 9 [15%]; *p* = 0.401) with the chi-squared test.

## 4. Discussion

### 4.1. Principal Findings

In this retrospective cohort study, no statistically significant differences in postoperative outcomes were detected between buccal sulcus and gingival margin incisions for mandibular fracture access at a level I major trauma centre. As the study was not designed as an equivalence or non-inferiority study, and the number of outcome events was small, the absence of a statistically significant difference does not establish that the two incisions were equivalent. No statistically significant difference was found between the buccal sulcus and gingival margin incision groups for postoperative infection, return to theatre (for any reason, for infection, or for malocclusion), wound dehiscence, or new numbness or paraesthesia (Table 4). These results provide a basis to consider future intraoral incision selection protocols at this institution, though they must be interpreted in the context of methodological limitations. Hence, these results are best interpreted as hypothesis-generating institutional data rather than a definitive comparison of the two incision types. It can help to define the effect size of future prospective trials and has identified the confounders that these trials would need to control for.

### 4.2. Interpretation in the Context of Existing Literature

A randomised controlled trial (RCT) conducted by Balasubramanian et al. in 2019 [2] compared buccal sulcus and gingival margin incisions for mandibular body fractures. They found that the gingival margin group had a statistically significant reduction in postoperative swelling and neurovascular impairment compared to the buccal sulcus group [2]. In comparison, our data included various mandibular fracture locations and did not replicate this finding in neurosensory outcomes. Whilst the rates of new numbness or paraesthesia were reduced in our buccal sulcus approach group, this was not statistically significant (*p* = 0.442) as shown in Table 4.

This discrepancy likely reflects differences in study design. The study by Balasubramanian et al. was a prospective RCT [2], with minimal selection bias, whereas our study was a retrospective cohort analysis where confounders were present. There were also differences in the techniques used for neurosensory outcome measurement. The RCT used specific pin-prick pain and direction sense of touch [2], whilst our study relied on the documentation of patient-reported symptoms in clinic notes.

Furthermore, a review by Chittoria et al. discusses a reduced risk of nerve injury and easier plate placement as advantages of the gingival margin incision [1]. Our study did not find a significant reduction in nerve injury. The data showed that a slightly higher proportion of the gingival margin group developed new paraesthesia, although this was not significant.

The buccal sulcus incision, whilst conventional, requires careful dissection to identify and protect the mental nerve [2]. In contrast, the gingival margin incision provides a more direct sub-periosteal plane to bone [1], which can simplify mental nerve isolation and minimise traction-related neuropraxia [2]. The fact that our study was unable to demonstrate this benefit may be attributed to its confounding factors.

### 4.3. Confounders

#### 4.3.1. Operator Variability

Operator variability is a consideration in this cohort. Fourteen surgeons of differing grades were primary operators and they were not assigned to a single incision group. Differences in operative experience, patient selection, fixation technique, postoperative management, and documentation may all have contributed to the observed outcomes. The retrospective design of this study makes it difficult to separate this operator variability.

Studies by Shetty et al. have shown that clinician-related characteristics, such as trauma load experience, influence their perception of mandibular fracture severity and the choice of treatment modality [3,4]. Similarly, Alfonso et al. found significant differences in operative and non-operative management of mandibular fractures between Oral and Maxillofacial Surgery, Plastic Surgery and Ear, Nose and Throat teams at the same level I major trauma centre. However, this did not translate to a difference in complication rates [5]. This highlights that operator variability has an impact on the fracture fixation choice, acting as a confounder.

#### 4.3.2. Choice of Osteosynthesis Plate and Position

There is a wide range of approaches to mandibular fracture fixation among surgeons and even within individual units. Although attempts have been made to standardise osteosynthesis approaches according to the principles outlined by Champy and Michelet [6,7], the heterogeneity of these fractures means that the choice and location of fixation have to be tailored to each patient. Inadequate fracture fixation or misplaced plates are also instigators of infection, and continued mobility at the fracture site predisposes to wound dehiscence; both of which may require a return to theatre and are independent of the incision employed. It is not possible to control for the multitude of osteosynthesis techniques used, even within just one hospital, in a retrospective analysis and is difficult to achieve even in prospective studies.

#### 4.3.3. Patient Demographics

Studies on the epidemiology of facial fractures found a male predominance, making up 60.1% [8] and 76.8% [9] of the cohort; this was higher in our cohort, with 93% male patients (Table 1). Despite logistic regression showing that the two incision groups were broadly comparable, two variables approached statistical significance: smoking (*p* = 0.071) and diabetes (*p* = 0.080). A higher proportion of smokers were in the buccal sulcus group, whilst all patients with diabetes were in the gingival margin group. Smoking is a well-established risk factor for wound healing complications, including infection [10] and dehiscence, by causing vasoconstriction and reducing immune function [11]. Likewise, the diabetic population has a higher risk of postoperative infection following mandibular fracture [12]. Whilst neither variable reached the threshold for statistical significance, the imbalance is clinically relevant [13] and may have confounded the rates of infection and wound dehiscence in our outcome data.

#### 4.3.4. Fracture Location

There were significant differences in mandibular fracture locations between the two incision groups. The gingival margin group had significantly more parasymphysis fractures (64%), and the buccal sulcus group had significantly more angle fractures (60%), as shown in Table 2. Subgroup analysis of outcomes per fracture location site was not possible due to the patient numbers in individual fracture locations being too small to support an adequately powered comparison, and several outcomes had zero values within these subgroups. Therefore, as the two incision groups represent substantially different fracture populations, the comparison of outcomes between them is accordingly exploratory and descriptive. Parasymphysis and angle fractures are associated with different risks and surgical challenges [14,15]. Fractures of the mandibular angle have the highest complication rates [15,16], likely due to its location on the mandible having unfavourable biomechanics and a greater influence by the muscles of mastication. Management of parasymphysis fractures requires close anatomic reduction to prevent malocclusion or widening of the lower facial third, which are both risks from this mandibular location [14].

Additionally, the buccal sulcus group had more comminution at the dentoalveolar level (Table 3) which alongside the increased angle fractures (Table 2), are more likely to have a tooth within the fracture line and hence require intra-operative extraction. Therefore, the higher extraction rate in the buccal sulcus group is more likely a consequence of the fracture pattern than the incision type chosen.

Different rates of inferior alveolar nerve dysfunction (IAND) are found for each mandibular fracture location [17]. This is due to the proximity of the fracture to the mental foramen, which must be carefully avoided during dissection [14]. A cohort study by Chandan et al. found IAND rates of 95.9% in body fractures, 90.1% in angle fractures and 27.6% in symphysis fractures [17]. The high rate of angle fractures is compounded by initial fracture displacement, which is a significant predictor of IAND [18]. A transbuccal trocar-assisted approach in combination with an intraoral incision can be used for angle fractures, but not parasymphysis fractures. The trocar-assisted approach has been linked with a statistically significant increased risk of postoperative nerve dysfunction [19]. Hence, fracture location is a direct confounder for neurosensory outcomes.

Infection risk may also vary by anatomical site. Malanchuk and Kopchak found that an angular tooth-bearing location of mandibular fractures contributes to postoperative infection rates [20]. Differences in fracture distribution in our incision groups suggest that the incision choice was likely dictated by fracture anatomy, a known determinant of surgical decision making [19].

### 4.4. Periodontal Health and Gingival Margin Incisions

A theoretical concern of impaired periodontal health from gingival margin incisions due to disruption of the gingival attachment exists, particularly in patients with pre-existing periodontal disease. Siddiqui et al. investigated this outcome prospectively and concluded that a well-performed gingival margin incision for mandibular fracture fixation did not affect periodontal health, with outcomes similar to the buccal sulcus incision [21]. A further consideration specific to gingival margin incisions is their predisposition to gingival recession. This is particularly relevant in the anterior aesthetic zone, which was where the gingival margin incision was used most commonly for parasymphysis fractures in this cohort (Table 2).

Periodontal disease is an important consideration in trauma patients and it is acknowledged that periodontal status was not assessed in this cohort. A baseline periodontal assessment did not form part of the acute maxillofacial trauma work-up in this department, which is similarly reflected across other centres in the UK. This highlights that routine preoperative periodontal screening is a priority for prospective studies and trauma protocols.

Periodontal health also impacts fracture healing. Multiple studies have established periodontal disease to be a risk factor for developing surgical site infections following mandibular fracture repair [11,22]. Janaphan et al. found that patients with stage 3 and 4 periodontitis had a postoperative wound infection risk that was over seven times greater than for patients without periodontitis [22]. Additionally, placement of intermaxillary fixation devices can initiate or progress periodontal disease due to poor oral hygiene and gingival trauma from wires and arch bars [23].

### 4.5. Strengths and Limitations

The main strength of this study is the volume of data analysed, which adds to the body of literature on incision choice in mandibular fracture repair. Our cohort of 138 patients had mandibular fractures at various locations and underwent surgical repair with an intraoral approach over a defined two-year period in a level I major trauma centre. The study assessed a range of important clinical outcomes, including return to theatre, infection, wound dehiscence, and new numbness or paraesthesia. By transparently reporting neutral outcomes, this study contributes to a growing evidence base.

However, this study is subject to several limitations. Firstly, the retrospective design and non-randomised allocation of patients to incision groups are sources of confounding factors, including operator variability, patient demographics and fracture location, which were earlier discussed. These factors limit the internal validity of the study when comparing the two incision techniques. Secondly, other clinically relevant variables, including antibiotic protocol, fixation method, the number and position of plates, use of a transbuccal approach, and whether fractures were open or closed, were not included in the analysis of this retrospective study. Each of these may influence infection, nerve dysfunction, malocclusion, and return to theatre. Hence, their absence limits the interpretation of the outcome comparisons.

Thirdly, the lack of statistically significant findings could either indicate that the incision groups have equivalent outcomes or that the study was underpowered to detect a true difference. A formal a priori power calculation was not performed in this retrospective study. Post hoc power estimation suggests that the sample size of 138 patients was below the study’s detectable effect size. For example, detecting the observed difference in return to theatre rates (14% vs. 10%) with 80% power would require approximately 2000 patients, numbers which were beyond the scope and capabilities of this study. Fourthly, our study lacks a standardised protocol to assess for numbness or paraesthesia, such as that used by Balasubramanian et al. [2]. Finally, assessment of baseline periodontal health was not recorded in trauma assessments, which could present an area for future prospective research.

## 5. Conclusions

This study found no statistically significant difference in postoperative outcomes between gingival margin and buccal sulcus incisions for mandibular fracture ORIF at a level I major trauma centre. The null hypothesis could not be rejected across all primary and secondary outcome measures. These findings are exploratory and descriptive. They do not establish equivalence between the two approaches, as the incision groups had significantly different fracture distributions. Both incisions were used by most of the operating surgeons.

A prospective multicentre randomized controlled trial restricted to a single, clearly defined fracture pattern, such as isolated parasymphysis fractures, would provide a more valid comparison by reducing anatomical confounding. Such a design would require standardized fixation and perioperative protocols, multiple participating surgeons to separate the incision effect from the surgeon effect, formal neurosensory assessment and preoperative periodontal screening. There should only be inclusion of cases where both incisions are clinically appropriate. Such a trial could create a standardised intraoral incision protocol for trauma centres managing mandibular fractures. Until this evidence is available, incision choice should continue to be guided by fracture anatomy and surgeon experience. Our study provides preliminary exploratory and descriptive institutional data to inform that decision-making process.

## Figures and Tables

**Table 1:** Participant demographics.

Variable	Buccal Sulcus N = 78 (%)	Gingival Margin N = 60 (%)	OR	95% CI	*p*-Value
Age					
Age—median (interquartile range)	29 (23–38)	36 (26–45)	-	-	0.039 *
Gender					
Male (reference)	74 (95)	55 (92)	ref	-	-
Female	4 (5)	5 (8)	1.68	-	0.502 ^†^
Diabetes status					
Non-diabetic (reference)	78 (100)	57 (95)	ref	-	-
Diabetic	0 (0)	3 (5)	-	-	0.080 ^‡^
Smoking status					
Non-smoker (reference)	40 (51)	40 (67)	ref	-	-
Smoker	38 (49)	20 (33)	0.53	0.26–1.06	0.071
Vaping status					
Non-vaper (reference)	70 (90)	55 (92)	ref	-	-
Vaper	8 (10)	5 (8)	0.80	0.25–2.57	0.702
Alcohol status					
No alcohol (reference)	48 (62)	40 (67)	ref	-	-
Alcohol consumed	30 (38)	20 (33)	0.80	0.40–1.62	0.534
Recreational drug status					
No drugs (reference)	65 (83)	49 (82)	ref	-	-
Drugs consumed	13 (17)	11 (18)	1.12	0.46–2.72	0.798

OR = odds of being assigned to the gingival margin group. Wald test *p*-values throughout. * Age summarized as median (interquartile range) and compared with the Mann–Whitney U test, as it was not normally distributed in either group. ^†^ Cell frequencies fell below five for the gender comparison. Fisher’s exact test *p*-value is reported. ^‡^ Zero diabetics in the buccal sulcus group results in OR not estimable by standard logistic regression. Fisher’s exact test *p*-value is reported.

**Table 2:** Anatomical fracture location.

Fracture Location	Buccal Sulcus Individual Fractures = 99 ^‡^ (%) [N = 78]	Gingival Margin Individual Fractures = 69 ^‡^ (%) [N = 60]	Total Individual Fractures = 168 ^‡^ (%) [N = 138]	Statistical Test		*p*-Value
Symphysis	2 (2)	2 (3)	4 (2)	Fisher’s exact	-	1.000
Parasymphysis	28 (28)	44 (64)	72 (43)	χ^2^ (df)	20.91 (1)	<0.001
Body	9 (9)	6 (9)	15 (9)	χ^2^ (df)	0.008 (1)	0.930
Angle	59 (60)	15 (22)	74 (44)	χ^2^ (df)	23.64 (1)	<0.001
Ramus	1 (1)	2 (3)	3 (2)	Fisher’s exact	-	0.569

^‡^ In patients presenting with a combination of mandibular fractures, each individual fracture was included as a separate entry; hence, total fractures > N.

**Table 3:** Fracture and dental characteristics.

Characteristic	Buccal Sulcus N = 78 (%)	Gingival Margin N = 60 (%)
Fracture Comminution		
Dentoalveolar	13 (17)	5 (8)
Inferior border of mandible	10 (13)	18 (30)
All comminution	23 (29)	23 (38)
Fracture Displacement		
Displaced	65 (83)	45 (75)
Undisplaced	13 (17)	15 (25)
Dental Characteristics		
Tooth in fracture line	73 (94)	51 (85)
Tooth extracted intra-operatively	30 (38)	6 (10)
Tooth fractured by injury	2 (3)	0 (0)

**Table 4:** Primary and secondary outcome measures.

Outcome	Buccal Sulcus N = 78 (%)	Gingival Margin N = 60 (%)	Statistical Test		*p* Value
Primary outcome measures					
Postoperative infection	7 (9)	5 (8)	χ^2^ (df)	0.018 (1)	0.893
Wound dehiscence	1 (1)	2 (3)	Fisher’s exact	-	0.580
Secondary outcome measures					
Return to theatre	11 (14)	6 (10)	χ^2^ (df)	0.528 (1)	0.467
Return to theatre for infection	6 (8)	3 (5)	Fisher’s exact	-	0.731
Return to theatre for malocclusion	5 (6)	3 (5)	Fisher’s exact	-	1.000
New numbness/paraesthesia	25 (32)	23 (38)	χ^2^ (df)	0.590 (1)	0.442
Partial new numbness	11 (14)	9 (15)	χ^2^ (df)	0.022 (1)	0.882
Complete new numbness	6 (8)	5 (8)	χ^2^ (df)	0.019 (1)	0.890
New paraesthesia	8 (10)	9 (15)	χ^2^ (df)	0.706 (1)	0.401

## Data Availability

The original contributions presented in this study are included in the article. Further inquiries can be directed to the corresponding author.

## Abbreviations

The following abbreviations are used in this manuscript:

RCT	Randomised Controlled Trial
ORIF	Open Reduction and Internal Fixation
SPSS	Statistical Package for the Social Sciences
OR	Odds Ratio
CI	Confidence Intervals
IAND	Inferior Alveolar Nerve Dysfunction
